# The Expiry of Humira^®^ Market Exclusivity and the Entry of Adalimumab Biosimilars in Europe: An Overview of Pricing and National Policy Measures

**DOI:** 10.3389/fphar.2020.591134

**Published:** 2021-01-08

**Authors:** Evelien Moorkens, Brian Godman, Isabelle Huys, Iris Hoxha, Admir Malaj, Simon Keuerleber, Silvia Stockinger, Sarah Mörtenhuber, Maria Dimitrova, Konstantin Tachkov, Luka Vončina, Vera Vlahović Palčevski, Gnosia Achniotou, Juraj Slabý, Leona Popelková, Kateřina Kohoutová, Dorthe Bartels, Ott Laius, Jaana E. Martikainen, Gisbert W. Selke, Vasileios Kourafalos, Einar Magnússon, Rannveig Einarsdóttir, Roisín Adams, Roberta Joppi, Eleonora Allocati, Arianit Jakupi, Anita Viksna, Ieva Greičiūtė-Kuprijanov, Patricia Vella Bonanno, Vincent Suttorp, Øyvind Melien, Robert Plisko, Ileana Mardare, Dmitry Meshkov, Tanja Novakovic, Jurij Fürst, Corinne Zara, Vanda Marković-Peković, Nataša Grubiša, Gustaf Befrits, Robert Puckett, Arnold G. Vulto

**Affiliations:** ^1^ Department of Pharmaceutical and Pharmacological Sciences, KU Leuven, Leuven, Belgium; ^2^ Strathclyde Institute of Pharmacy and Biomedical Sciences, University of Strathclyde, Glasgow, United Kingdom; ^3^ Division of Public Health Pharmacy and Management, School of Pharmacy, Faculty of Health Sciences, Sefako Makgatho Health Sciences University, Pretoria, South Africa; ^4^ Division of Clinical Pharmacology, Department of Laboratory Medicine, Karolinska Institutet, Karolinska University Hospital Huddinge, Stockholm, Sweden; ^5^ Department of Pharmacy, University of Medicine Tirana, Tirana, Albania; ^6^ Austrian Social Insurance, Vienna, Austria; ^7^ Faculty of Pharmacy, Medical University of Sofia, Sofia, Bulgaria; ^8^ Faculty of Health Studies, University of Rijeka, Rijeka, Croatia; ^9^ Health Insurance Organization (HIO), Nicosia, Cyprus; ^10^ State Institute for Drug Control, Prague, Czechia; ^11^ Amgros, Copenhagen, Denmark; ^12^ State Agency of Medicines, Tartu, Estonia; ^13^ Pharmaceuticals Pricing Board, Ministry of Social Affairs and Health, Helsinki, Finland; ^14^ AOK Research Institute (WIdO), Berlin, Germany; ^15^ National Organization for the Provision of Healthcare Services (EOPYY), Athens, Greece; ^16^ Ministry of Welfare, Reykjavik, Iceland; ^17^ The National University Hospital of Iceland, Reykjavik, Iceland; ^18^ St James’s Hospital, Dublin, Ireland; ^19^ Clinical Research and Drug Evaluation Unit, Local Health Authority of Verona, Verona, Italy; ^20^ Mario Negri Institute for Pharmacological Research (IRCCS), Milan, Italy; ^21^ UBT – Higher Education Institute, Prishtina, Kosovo; ^22^ Department of Medicines and Medical Devices, The National Health Service, Riga, Latvia; ^23^ Ministry of Health of the Republic of Lithuania, Vilnius, Lithuania; ^24^ Erasmus University Medical Center, Rotterdam, Netherlands; ^25^ Reviews and Health Technology Assessments, Norwegian Institute of Public Health, Oslo, Norway; ^26^ HTA Consulting, Cracow, Poland; ^27^ Public Health and Management Department, Faculty of Medicine, “Carol Davila”, University of Medicine and Pharmacy Bucharest, Bucharest, Romania; ^28^ V. A. Trapeznikov Institute of Control Sciences of Russian Academy of Sciences, Moscow, Russia; ^29^ ZEM Solutions, Belgrade, Serbia; ^30^ Health Insurance Institute, Ljubljana, Slovenia; ^31^ Drug Area, Catalan Health Service, Barcelona, Spain; ^32^ Department of Social Pharmacy and Pharmacy Practice, Faculty of Medicine, University of Banja Luka, Banja Luka, Bosnia and Herzegovina; ^33^ Health Insurance Fund, Banja Luka, Bosnia and Herzegovina; ^34^ Stockholm County Council, Stockholm, Sweden; ^35^ NHS Greater Glasgow and Clyde, Queen Elizabeth University Hospital, Glasgow, United Kingdom

**Keywords:** adalimumab, prices, biosimilars, Europe, Humira, competition, health policy

## Abstract

**Background:** From October 2018, adalimumab biosimilars could enter the European market. However, in some countries, such as Netherlands, high discounts reported for the originator product may have influenced biosimilar entry.

**Objectives:** The aim of this paper is to provide a European overview of (list) prices of originator adalimumab, before and after loss of exclusivity; to report changes in the reimbursement status of adalimumab products; and discuss relevant policy measures.

**Methods:** Experts in European countries received a survey consisting of three parts: 1) general financing/co-payment of medicines, 2) reimbursement status and prices of originator adalimumab, and availability of biosimilars, and 3) policy measures related to the use of adalimumab.

**Results:** In May 2019, adalimumab biosimilars were available in 24 of the 30 countries surveyed. Following introduction of adalimumab biosimilars, a number of countries have made changes in relation to the reimbursement status of adalimumab products. Originator adalimumab list prices varied between countries by a factor of 2.8 before and 4.1 after loss of exclusivity. Overall, list prices of originator adalimumab decreased after loss of exclusivity, although for 13 countries list prices were unchanged. When reported, discounts/rebates on originator adalimumab after loss of exclusivity ranged from 0% to approximately 26% (Romania), 60% (Poland), 80% (Denmark, Italy, Norway), and 80–90% (Netherlands), leading to actual prices per pen or syringe between €412 (Finland) and €50 – €99 (Netherlands). To leverage competition following entry of biosimilar adalimumab, only a few countries adopted measures specifically for adalimumab in addition to general policies regarding biosimilars. In some countries, a strategy was implemented even before loss of exclusivity (Denmark, Scotland), while others did not report specific measures.

**Conclusion:** Even though originator adalimumab is the highest selling product in the world, few countries have implemented specific policies and practices for (biosimilar) adalimumab. Countries with biosimilars on the market seem to have competition lowering list or actual prices. Reported discounts varied widely between countries.

## Introduction

Biological medicines represent a multi-billion-dollar medicines’ industry used in the treatment of cancer and autoimmune diseases. They represent a challenge for healthcare budgets due to their often high prices and increasing use (Moorkens et al., 2016; Godman et al., 2018; La Merie, 2018; IQVIA, 2019). Within the biopharmaceutical market, the global tumor necrosis factor-alpha (TNF-α) inhibitors market (adalimumab, infliximab, etanercept, certolizumab pegol and golimumab) was worth US$40 billion in 2018, with half of these sales coming from one product, Humira^®^ (adalimumab) (Grand View Research, 2019). With sales of US$20 billion in 2018, Humira^®^ is the highest selling product in the world and generates 60% of AbbVie’s revenue (AbbVie News Center, 2019). Consequently, AbbVie has used an extensive strategy (related to patent filing, marketing, and pricing) to protect the sales and revenue of Humira^®^ from competitor products (Mukherjee, 2019; Hordijk, 2019; Dunn, 2019; Sagonowsky, 2017).

On October 16, 2018, product-specific patent protection on Humira^®^ (extended with a supplementary protection certificate and an additional 6 months of pediatric exclusivity) came to an end in most European Union (EU) countries, 15 years after the initial marketing authorization (Storz, 2017). However, subsequent (‘secondary’) patents for dosage regimens for rheumatoid arthritis and inflammatory bowel disease were still active in Europe (Moorkens et al., 2020). In addition, in the United States (US) uncertainty about infringement on formulation and dosage patents existed (Storz, 2017; Holman, 2017). To address this uncertainty, biosimilar developers started several patent litigation cases against AbbVie for these secondary patents. Eventually eight companies developing biosimilars reached a settlement agreement that consists of three parts: 1) the biosimilar companies will not enter the United States market before 2023, 2) biosimilars are allowed access to the EU market as of October 2018 without risk of litigation, and 3) an undisclosed royalty arrangement (Dunn, 2019; AbbVie, 2018; AbbVie, 2019). In more detail: the companies will each pay royalties to AbbVie for a non-exclusive licensing agreement on EU patents related to Humira^®^ starting from October 16, 2018 and United States patents starting between January 31, 2023 and November 20, 2023, depending on company-specific arrangements. These arrangements provide AbbVie with a monopoly on its largest market, the US, until 2023 (Dunn, 2019; AbbVie, 2019). For Europe, from October 16, 2018 adalimumab biosimilars could potentially enter the market and offer countries, by increasing competition, new ways to reduce expenditures in immunological disease areas using TNF-α inhibitors. Table 1 shows that several adalimumab biosimilars were already approved by October 2018 by the European Medicines Agency (EMA) and could, after the completion of pricing and reimbursement procedures, immediately enter the market; others were approved later (European Medicines Agency, 2020).

**TABLE 1:** Adalimumab biosimilars approved by the European Medicines Agency for use in the European Union as of September 2020 (European Medicines Agency, 2020).

Brand name	Marketing authorization holder	Marketing authorization date
Amgevita^®^	Amgen	March 21, 2017
Imraldi^®^	Samsung Bioepis	August 24, 2017
Hyrimoz^®^/Hefiya^®^/Halimatoz^®^ ^a^	Sandoz	July 26, 2018
Hulio^®^	Mylan	September 16, 2018
Idacio^®^	Fresenius Kabi	April 02, 2019
Amsparity^®^	Pfizer	February 13, 2020

a Different brand names might be used as a marketing strategy or to circumvent potential patent issues via different indications on the label.

The increasing use of biosimilars is important across Europe with global expenditure on medicines likely to reach US$1.5trillion by 2023, driven by increasing use of biological medicines (IQVIA, 2019), and resources saved from their increasing use can help fund increased use of medicines for patients with non-communicable diseases with aging populations as well as new premium priced medicines (Inotai et al., 2017; Povero and Pradelli, 2020). In addition, enhance the opportunity for greater access to effective biological medicines among Central and Eastern European (CEE) countries as well as other lower- and middle-income countries where access has been an issue due to high prices, sustainability of healthcare systems, and high co-payment levels (Mihajlo, 2014; Putrik et al., 2014; Jakovljevic et al., 2015; Kovacevic et al., 2015; Pichon-Riviere et al., 2015; Baumgart et al., 2019). We are aware that expenditure on healthcare has been rising steadily among middle-income countries including the BRICS (Brazil, Russia India, China and South Africa), with growth rates exceeding those among high income countries in recent years (Jakovljevic, 2016; Jakovljevic and Getzen, 2016; Jakovljevic et al., 2020), with a similar difference seen between CEE and Western European countries (Jakovljevic et al., 2016). However, there are continuing concerns with high co-payment levels, and reimbursement issues with high prices, among BRICS and other similar countries, which will adversely impact on the access and use of biologicals unless their costs appreciably fall (Jakovljevic et al., 2017; Kastor and Mohanty, 2018; Jakovljevic et al., 2019; Jakovljevic et al., 2019; Jakovljevic et al., 2020).

With the market entry of adalimumab biosimilars, discounts of up to 89% on Humira^®^ were reported in the Netherlands, ensuring that after 1 year 70% of patients were still using the originator product (Hordijk, 2019). This triggered the authors to study whether this situation also occurs in other European countries and how countries react to these new price dynamics. This level of discount will undoubtedly enhance availability and access to biological medicines among CEE countries.

Consequently, the aim of this paper is to provide an overview of (list) prices and reimbursement status of originator adalimumab in different European countries, before and after the entry of adalimumab biosimilars to the market, and study how countries respond to this price evolution by implementing policy measures relevant to the use of adalimumab. The authors also discuss the strategy of the originator company and provide future guidance, especially with respect to potential moves by other manufacturers of originator biologicals to offer appreciable discounts to limit the attractiveness of the biosimilars’ market.

A discussion of general policy measures and practices regarding biosimilars in each country is considered out of the scope of this paper. Several papers have already mapped policies for biosimilars on a national or regional/local level (Renwick et al., 2016; Moorkens et al., 2017; Rémuzat et al., 2017a; Rémuzat et al., 2017b; Moorkens et al., 2019a; Moorkens et al., 2019b; Kim et al., 2020; Moorkens et al., 2020), although the market is rapidly evolving. This paper reflects some of these changes.

## Methods

The overview of prices, reimbursement status and policy measures for originator adalimumab and biosimilars was obtained using a survey distributed via email amongst members of the Piperska Group. This group is a Pan-European network of health authority, health insurance company personnel and their advisers, as well as academics performing research on the rational use of medicines. The group’s research includes ways to enhance the use of low-cost generics and biosimilars, and development of new models to optimize the managed entry of new medicines, including potential managed entry agreements. The group also debates key issues, including adaptive pathways and a single Health Technology Assessment (HTA) body for Europe (Moorkens et al., 2017; Piperska Group, 2015; Godman et al., 2010; Moon et al., 2014; Malmstrom et al., 2013; Godman et al., 2012; Vella Bonanno et al., 2017; Vella Bonanno et al., 2019; Ferrario et al., 2017; Godman et al., 2019). No approval from a research ethics committee was required for this study.

The survey consisted of three parts. The intention being to assess how the healthcare system in different European countries is organized (especially related to biological medicines), how the adalimumab market is evolving, and how countries have responded to these changes, especially regarding any activities from AbbVie to appreciably lower the price of originator adalimumab. Firstly, more general information on the financing of medicines in each country and the level of co-payment for biological medicines was requested. Secondly, the availability of biosimilars, the reimbursement status of originator adalimumab, listed and reimbursed prices, and potential discounts, before and after loss of exclusivity (up to May 10, 2019), were requested. The 40 mg syringe or pen was chosen as a reference for price comparison, as for this dosage all adalimumab products have a marketing authorization. Finally, respondents were asked to elaborate on health authority/health insurance company measures related to the use of adalimumab in their country, especially measures affecting the utilization of biosimilars since the perspective of this paper is that of health authorities. The survey was written in English and included both open- and closed-ended questions.

Data was collected between May and November 2019. One or more senior-level health authority personnel or their advisers were approached in each country for their help. We have used this approach before when analyzing the influence of different initiatives and pricing policies to provide robust information (Godman et al., 2010; Baumgärtel et al., 2012; Godman et al., 2013; Godman et al., 2014a; Godman et al., 2014b; Moon et al., 2014; Ferrario et al., 2017; Moorkens et al., 2017; Godman et al., 2019). The survey responses were analyzed descriptively, with open-ended questions examined in a qualitative way. Prices were typically list prices including value added tax (VAT) and margins (where appropriate). Prices in different currencies were converted to euros with the exchange rate of December 19, 2019 for Bulgaria, the Czech Republic, Denmark, Iceland, Norway, Poland, Romania, Scotland, and Sweden. Some countries already provided prices in euro, but have a different currency (Albania, Croatia, Serbia, Republic of Srpska, Russia). Discounts were not always available on a national level for some countries and can pertain to a single region or hospital group, e.g., the Veneto region in Italy, and hospital groups in the Netherlands. When this is the case, it is indicated in the text. The country experts were asked to critically review the paper and correct any potential errors in the interpretation of their responses prior to submission.

## Results

Completed surveys were obtained from 30 countries, 23 EU Member States, plus Albania, Iceland (member of the European Economic Area (EEA)), Norway (member of the EEA), Russia, Serbia, the Republic of Kosovo, and the Republic of Srpska (entity in Bosnia and Herzegovina). Information on financing of medicines in the different countries surveyed and levels of co-payment, gathered via part 1 of the survey, can be found in the Supporting Material. Results from part 2 and 3 of the survey on the availability of biosimilars and pricing, reimbursement and policy measures related to adalimumab are discussed in the following sections. While we used the EMA definition of a biosimilar (EMA, 2014), we also considered similar products not regulated by the EMA to be relevant for our analysis.

### Availability of Adalimumab Biosimilars

In May 2019, biosimilars for adalimumab were available in 24 of the 30 countries surveyed (Table 2). Countries with no adalimumab biosimilars on the market at that time were Cyprus, Greece, Kosovo, Malta, Serbia and the Republic of Srpska; with Greece at the time waiting for approval of reimbursement (eventually received in July 2020). In Russia, only a non-EMA-approved biosimilar was available, i.e., Dalibra^®^ (marketed by the Russian biotechnology company BIOCAD). The adalimumab biosimilar Idacio^®^, which was given marketing authorization in April 2019, was only just launched in Germany (May 1, 2019).

**TABLE 2:** Overview of availability of originator adalimumab and biosimilars in the different European countries surveyed (as of May 10, 2019).

Country	Originator adalimumab	Amgevita^®^	Hulio^®^	Hyrimoz^®^/Hefiya^®^/Halimatoz^®^	Imraldi^®^	Idacio^®^/Kromeya^®^	Other
Albania	X	—	X	—	—	—	—
Austria	X	X	X	X (Hyrimoz^®^)	X	—	—
Belgium	X	X	X	X (Hyrimoz^®^)	X	—	—
Bulgaria	X	X	X	X (Hyrimoz^®^)	—	—	—
Croatia	X	X	X	X (Hyrimoz^®^)	X	—	—
Cyprus	X	—	—	—	—	—	—
Czech rep	X	X	X	X (Hyrimoz^®^)	X	—	—
Denmark	X	X	-_a_	X (Hyrimoz^®^)	X	—	—
Estonia	X	—	X	—	X	—	—
Finland	X	X	X	X (Hyrimoz^®^)	—	—	—
Germany	X	X	X	X (Hyrimoz^®^)	X	X (Idacio^®^)	—
Greece	X	- (Not yet reimbursed)°	- (Not yet reimbursed)°	- (Not yet reimbursed)°	- (Not yet reimbursed)°	-°	—
Iceland	X	—	—	—	X	—	—
Ireland	X	X	X	—	X	—	—
Italy	X	X	—	X (Hyrimoz^®^)	X	—	—
Kosovo	—	—	—	—	—	—	—
Latvia	X	—	—	X (Hyrimoz^®^)	—	—	—
Lithuania	X	X	X	—	—	—	—
Malta	X	—	—	—	—	—	—
Netherlands	X	X	X	X (Hyrimoz^®^)	X	—	—
Norway	X	X	X	X (Hyrimoz^®^)	X	—	—
Poland	X	X	—	X (Hyrimoz^®^)	X	—	—
Romania	X	X	X	X (Hyrimoz^®^)	—	—	—
Russia	X	—	—	—	—	—	X (Dalibra^®^)
Scotland	X	X	X	X (Hyrimoz^®^)	X	—	—
Serbia	X	—	—	—	—	—	—
Slovenia	X	X	X	X (Hyrimoz^®^)	X	—	—
Spain	X	X	X	X (Hyrimoz^®^)	X	—	—
Srpska	X	—	—	—	—	—	—
Sweden	X	X	X	X (Hyrimoz^®^)	X	—	—

X: Available (i.e. patient access via reimbursement/funding).

-: Not available to patients.

a Product might be on the market and reimbursed, but not first-choice in the recommended ranking.

°: Reimbursed since July 2020.

### Reimbursement Status and Treatment Setting of Originator Adalimumab

In all of the countries surveyed, originator adalimumab is reimbursed (fully or partially, and sometimes with restrictions in use), except for Kosovo, where it is not marketed. More information on co-payment for adalimumab products in each of the countries surveyed can be found in the Supporting Material. In 15 of the countries surveyed, adalimumab can be dispensed in ambulatory care (retail pharmacy or similar setting), while in 14 countries its use is restricted to hospital use only (including day care/ambulant patients) (Table 3).

**TABLE 3:** Overview of reimbursement status and treatment setting of originator adalimumab (Humira^®^) in the different European countries surveyed (May 2019).

Country	Reimbursement of originator adalimumab (May 10, 2019)	
Albania	H	Yes, in the hospital setting since 2016 and when prescribed according to the protocol. Reimbursement price same for originator and biosimilar, but due to higher list price for originator, co-payment higher (€108 patient co-payment originator vs. €12 for the biosimilar).
Austria	AC	Yes, in ambulatory care. Some restrictions have been lifted after the introduction of biosimilars.
Belgium	AC	Yes, mainly in ambulatory care and sometimes in the hospital setting.
Bulgaria	AC	Yes, in ambulatory care. When meeting specific criteria, 75% reimbursement for rheumatoid arthritis, Crohn’s disease, ulcerative colitis, and psoriasis. Internal reference price has decreased since entry of biosimilars, resulting in increased co-payment for patients.
Croatia	H	Yes, in ambulatory care and hospital setting, with prescribing restrictions. Restrictions not changed despite the entry of biosimilars.
Cyprus	H	Yes, in the hospital setting.
Czech rep	H	Yes, mainly for ambulant patients in the hospital and sometimes in the inpatient setting.
Denmark	H	Yes, in the hospital setting. Medicines are ranked according to price. Use of the biosimilar is recommended.
Estonia	H	Yes, in the hospital setting. Following the entry of biosimilars, from July 2019, both in ambulatory care and hospital setting.
Finland	AC	Yes, in ambulatory care and hospital setting. In ambulatory care, reimbursement is restricted to patients who had inadequate response to conventional treatment.
Germany	AC	Yes, in ambulatory care and hospital setting.
Greece	AC	Yes, in ambulatory care via EOPYY’s pharmacies (public pharmacies).
Iceland	H	Yes, in the hospital setting when there is an acceptable clinical reason to use Humira^®^ instead of Imraldi^®^ (a less expensive adalimumab biosimilar).
Ireland	AC	Yes, in ambulatory care. However, reimbursement status/level may be subject to additional controls following the entry of biosimilars and introduction of a ‘Best value biological policy’. Originator adalimumab is not included as a best value biological.
Italy	H	Yes, in the hospital setting.
Kosovo		Humira^®^ is not marketed in Kosovo.
Latvia	AC	Yes, in ambulatory care. Reimbursement status has not changed since the entry of biosimilars, but internal reference price has decreased to the price of the biosimilar.
Lithuania	AC	Yes, in ambulatory care when the least expensive TNF-α inhibitors are contraindicated or treatment is not effective.
Malta	AC	Yes, in ambulatory care. Adalimumab is provided through the national health services on a named-patient basis to patients who fulfill criteria for entitlement to free medicines and the specific indications for which adalimumab is indicated. List of patients taking the drug is maintained and treatment is monitored. This has not changed since the entry of biosimilars. In the new procurement cycle, an adalimumab biosimilar has won (September 2019).
Netherlands	H	Yes, in the hospital setting. The height of the add-on (a financial supplement that hospitals declare directly to the health insurer) has been lowered since the entry of biosimilars.
Norway	H	Yes, in the hospital setting. Medicines are ranked according to price. Use of originator adalimumab is recommended.
Poland	H	Yes, in the hospital setting via a specific drug program (list B).
Romania	AC	Yes, prescribed in ambulatory care (retail budget for prescriptions) and administered/monitored in ambulatory care or day hospitalization (if necessary). Originator adalimumab had first no co-payment, but this was increased to €106.3 per unit following a change in the reference price after the entry of biosimilars.
Russia	AC	Yes, in ambulatory and hospital care according to a number of federal programs.
Scotland	AC	Yes, via homecare services, but can be used in the hospital setting.
Serbia	H	Yes, in the hospital setting, with prescribing restrictions.
Slovenia	AC	Yes, mainly in ambulatory care, but can be used in the hospital setting.
Spain	H	Yes, in the hospital setting.
Srpska	H	Yes, in the hospital setting, with prescribing restrictions.
Sweden	AC	Yes, in ambulatory care and hospital setting.

H: Hospital setting only; AC: Also available in ambulatory care.

Following the entry of adalimumab biosimilars, few countries have been able to make changes to the reimbursement status/level or treatment setting where adalimumab is available. For example, in Austria restrictions for prescribing of adalimumab have been lifted. Originally, originator adalimumab was listed in the Dark Yellow Box, meaning it could only be reimbursed after prior approval by the chief medical officer of the respective health insurance fund (Godman et al., 2009). Reimbursement was limited to certain criteria regarding disease group, failure of pre-treatments and prescription by specialized physicians. However, adalimumab biosimilars qualified for the Green Box status (automatic reimbursement, i.e., approval by the chief medical officer is not required), albeit subject to certain criteria. Price negotiations with AbbVie eventually led to the same arrangement for originator adalimumab, i.e. Green Box assignment, following agreed price reductions. In Iceland, originator adalimumab is not recommended as a first-choice medicine to treat immunological conditions, since less expensive biosimilars are available, and is only reimbursed when the prescriber provides specific medical reasons. In Ireland, the introduction of the ‘Best value biological policy’ has resulted in recommendations for biosimilar adalimumab (with or without citric acid) and biosimilar etanercept to be prescribed as first line treatments when TNF-α inhibitors are warranted, as well as helping with switching (HSE Ireland - Clinical Stratgey and Programmes Division, 2019a; HSE Ireland - Clinical Stratgey and Programmes Division, 2019b). In some countries, the internal reference price has decreased to the price level of the biosimilar (or even lower), resulting in an increased co-payment for patients on the originator product if the price of the originator product exceeds the reference price (Bulgaria, Latvia, and Romania). However, in Latvia, co-payments for originator adalimumab are minimal after a confidential reduced price was offered. In the Czech Republic, patient co-payment for originator adalimumab is higher than for the biosimilars; however, co-payments can be lowered when originator adalimumab is included in a contract between the marketing authorization holder and health insurance companies, or hospitals can also fully cover outpatient care. In Estonia, a change in setting from hospital only to both ambulatory care and hospital setting was introduced following the entry of adalimumab biosimilars.

### Price Evolution of Originator Adalimumab

#### Situation Before Loss of Exclusivity

A large variation in list prices can be observed for originator adalimumab (40 mg syringe or pen) in the countries surveyed before loss of exclusivity (Table 4). When list prices were reported (25/30 countries, excluding Cyprus, Estonia, Kosovo, Russia and the Republic of Srpska), these ranged from €335.5 per unit in Slovenia to €955.47 in Germany (factor 2.8 difference). On average, the list price was €516 per unit, with a median of €500 per unit.

**TABLE 4:** Evolution of originator adalimumab (Humira^®^) prices (40 mg syringe or pen), presented as price before and after loss of exclusivity (up to May 10, 2019), for the different European countries surveyed. Unless indicated otherwise, the presented prices are list prices including value added tax and margins (where appropriate).

Country	Price of originator adalimumab before loss of exclusivity	Price of originator adalimumab after loss of exclusivity
Albania	2014 – March 2019: €580.25 per unit (€1,160.5 per package of 2). No confidential discounts or rebates exist.	From May 2019: €335.49 per unit (€670.98 per package of 2). No confidential discounts or rebates exist.
		The biosimilar Hulio^®^ is listed at €253 per unit and thus recommended as first choice.
Austria	€479.88 per unit (€959.75 per package of 2) Ex-factory price: €450 per unit (€900 per package of 2). No confidential discounts exist.	€233.65 (€467.30 per package of 2) Ex-factory price: €213 per unit (€426 per package of 2). Further confidential discounts exist.
		In May 2019, the least expensive biosimilar was Hulio^®^, with a list price of €220.13 per unit, plus a confidential rebate.
		From September 2019, the price of Humira^®^ further decreased to €199.35 per unit, plus a confidential rebate. In Austria, when a third biosimilar enters the market, all biosimilars and the originator listed in reimbursement need to adjust their price to the third biosimilar. This adjustment for all products happened in September 2019.
		As of November 2019, Idacio^®^ is the least expensive adalimumab product, listed at €199.3, plus a confidential rebate.
Belgium	February 1, 2018: €522 per unit (€1,043.75 per package of 2); €518 per unit (€3,107.47 per package of 6).	January 2019: €359 per unit (€717.74 per package of 2); €338 per unit (€2,025.86 per package of 6).
		May 2019: €315.25 per unit (€630.5 per package of 2); €296.26 per unit (€1,777.55 per package of 6).
	October 1, 2018: €518 per unit (€1,036.91 per package of 2); €515 per unit (€3,087.02 per package of 6).	Price reductions are the result of mandatory decreases. No confidential discounts or rebates exist.
	Humira^®^ was included in a confidential managed entry agreement.	Biosimilars are priced somewhat lower: €291.43 per unit (€582.85 per package of 2); €288.34 per unit (€1,730.06 per package of 6).
Bulgaria	Up to October 2018: €409.48 per unit (€818.95 per package of 2). Confidential discounts may apply.	List prices have been unchanged. Confidential discounts may apply.
		The available adalimumab biosimilars have a lower price.
Croatia	From February to June 2017: €509.19 per unit.	The list price has been unchanged.
	From July 2017 to May 2018: €502.1 per unit.	Biosimilars are listed at €412.24 per unit (Amgevita^®^), €371.02 per unit (Imraldi^®^ and Hyrimoz^®^), and €333.92 per unit (Hulio^®^).
	From Augustus 2018 to March 2019: €484.99 per unit.	From June 2019: €333.92 per unit for the originator and biosimilars.
	The product was under an undisclosed managed entry agreement.	The managed entry agreement should still be in place.
Cyprus	Humira^®^ is procured by the ministry of health through public procurement. Actual prices are confidential.	Humira^®^ is procured by the ministry of health through public procurement. Actual prices are confidential.
Czech rep	2014:	Maximum price: €517.28 per unit (€1,034.55 per package of 2).
	Maximum price: €537.57 per unit (€1,075.14 per package of 2).	Reimbursement price: €416.10 per unit (€832.19 per package of 2).
	Reimbursement price: €505.84 per unit (€1,011.67 per package of 2).	
	2018:	From June 2019: €338.16 per unit, reimbursed up to €237.32 per unit.
	Maximum price: €524.90 per unit (€1,049.79 per package of 2).	
	Reimbursement price: €381.25 per unit (€762.49 per package of 2).	The least expensive products to use are biosimilars Amgevita^®^ and Hulio^®^. Since July and August 2019, respectively, they have no co-payment.
	Confidential contracts can exist between hospitals and marketing authorization holders, or between health insurance companies and marketing authorization holders.	Confidential contracts can exist between hospitals and marketing authorization holders, or between health insurance companies and marketing authorization holders, for both originator and biosimilar products.
Denmark	October 2018: €518.40 per unit (€1,036.79 per package of 2).	List prices for the originator have been unchanged. Discounts of more than 80% have been reported.
	There were no additional discounts or rebates.	In a multi-winner tender two biosimilars (Imraldi^®^, Hyrimoz^®^), plus Humira^®^, were chosen and also one player for the pediatric form (Amgevita^®^).
		Biosimilars are listed at a lower price, but actual prices are comparable with the actual price for Humira^®^.
Estonia	Humira^®^ is procured by hospitals. Prices are not known.	Humira^®^ is, from July 2019, also available in ambulatory care: ex-pharmacy. price, including 9% VAT, of adalimumab regardless of brand name is €125.5 per unit (€251 per package of 2).
		There are no additional discounts or rebates.
Finland	Retail price with VAT: €580 per unit (€1,160.30 per package of 2). Wholesale price: €463 per unit (€925.61 per package of 2). There were no additional discounts or rebates.	Retail price with VAT: €412 per unit (€824.31 per package of 2).
	For the hospital setting, actual prices are not known.	Wholesale price: €324 per unit (€647.93 per package of 2).
		There are no additional discounts or rebates.
		The adalimumab biosimilars Amgevita^®^ and Hulio^®^ are the least expensive treatment options for adalimumab.
Germany	Public list price in ambulatory sector: €955.47 per unit (€1911.74 per package of 2); with parallel imports down to €871.78 per unit (€1744 per package of 2). Rebate contracts exist between the pharmaceutical company and health insurance funds.	List prices for the originator have been unchanged. Price for biosimilars is generally €572.32 per unit. Rebate contracts exist between the pharmaceutical company and health insurance funds for both originator and biosimilar products. Tenders in the hospital setting are also still operational.
	For the hospital setting, actual prices are not known. There have been tenders (with discounts) for Humira^®^ since at least July 2016.	
Greece.	September 2018: Retail price €430.11 per syringe/€431.39 per pen; wholesale price: €368.87 per syringe/€369.97 per pen, ex-factory price €363.42 per syringe/€364.50 per pen.	May 2019: List prices have been unchanged. There is not yet a special discount.
	For hospitals and EOPYY pharmacies, a hospital price is used that is a standard percentage of the wholesale price.	Biosimilars are listed at a lower retail price, but are not yet reimbursed: €344.59 per unit (Amgevita^®^), €337.84 per unit (Halimatoz^®^/Hefiya^®^/Hyrimoz^®^), €301.88/€269.58 per unit in package of 1/2 (Hulio^®^), €332.19 per unit (Imraldi^®^).
		July 2020: Confidential discounts are set for originator and biosimilar adalimumab after a negotiation procedure.
Iceland	October 2018: €525.49 per unit (€1,050.97 per package of 2); without 24% VAT: €423.78 per unit (€847.56 per package of 2). TNF-α inhibitors are purchased after a tender agreement, as is the case for all hospital medicines. No confidential discounts were offered for Humira^®^.	Official list price of Humira^®^ including VAT (24%) is €513.47 per unit (€1,026.94 per package of 2). Imraldi^®^ and Humira^®^ reimbursement prices are strictly confidential. The biosimilar Imraldi^®^ is the least expensive product to use.
Ireland	August 2016: €656.58 to €670 per unit (€1,313.15 to €1,339.99 per package of 2) inclusive of 8% wholesaler mark-up and 23% VAT; €541.3 to €552.36 (€1,082.60 to €1,104.72 per package of 2) inclusive of 8% wholesaler mark-up, exclusive of 23% VAT.	The framework agreement between government and industry specifies at least a 20% reduction from the list price of a biological and a 12.5% rebate, which overall equates to 30% mandated reduction, following the introduction of a biosimilar onto the Irish market. This is not a commercially confidential rebate.
	August 2018: €630.20 to €644.34 per unit (€1,260.40 to €1,288.68 per package of 2) inclusive of 8% wholesaler mark-up and 23% VAT; €519.56 to €531.21 per unit (€1,039.11 to €1,062.42 per package of 2) inclusive of 8% wholesaler mark-up, exclusive of 23% VAT.	From December 2018: €535.99 per unit (€1,071.98 per package of 2) inclusive of 8% wholesaler mark-up and 23% VAT; €441.89 per unit (€883.77 per package of 2) inclusive of 8% wholesaler mark-up, exclusive of 23% VAT. An additional 12.5% rebate is paid back to the payer by the manufacturer, i.e. €51.15 per unit.
	Information on discounts and/or rebates was not available.	Further confidential discounts are available for the recommended biosimilar products. As the originator was not chosen as one of the best value biologicals, it was reported that it would be unlikely that further discounts are available nationally.
Italy	Since 2016, €795.8 per unit (€1,591.60 per package of 2). Ex-factory price from 2016 onwards: €318.32 per unit.	List and ex-factory prices have been unchanged, but discounts on Humira^®^ of 50% have been reported (based on ex-factory prices used for tendering; 80% discount when calculated against the list price).
	Further confidential discounts existed.	Using centralized purchasing the Veneto region is paying: - Humira^®^ (to be used only in those patients who cannot be switched to the biosimilar): €160 per unit.
		- Amgevita^®^ (winner of the bid): €98.50 per unit.
Kosovo	Humira^®^ is not marketed in Kosovo and is also not on the national essential medicines list (NEML). However, from the group of TNF-α inhibitors, etanercept and infliximab were listed on the NEML.	Humira^®^ is still not marketed in Kosovo and is also not on the national essential medicines list (NEML). Etanercept and infliximab were again on the NEML.
Latvia	Pharmacy price including VAT: €462.64 per unit, wholesale price: €410.05 per unit.	List prices have been unchanged. After the biosimilar was included in the positive list, a confidential discount was offered to lower co-payment for patients on the originator product.
	There was a confidential discount.	The biosimilar is listed at a lower price.
Lithuania	List price: €380.86 per unit (€761.72 per package of 2), reimbursement price: €378.51 per unit (€757.01 for a package of 2).	List price: €263.86 per unit (€527.71 per package of 2), reimbursement list price: €261.5 per unit (€523.00 for a package of 2).
	There were further confidential discounts.	Confidential discounts exist. The marketing authorization holder also has the possibility to cover co-payment.
		From June 2018, the biosimilar should be priced 15% lower than the originator product (previously this was 30% lower for the first biosimilar, and an additional 15% for subsequent biosimilars). The biosimilar was the least expensive product.
Malta	List price: €500 per unit. There was further negotiation (result non-disclosed).	Since September 2019, Humira^®^ is no longer procured. All patients will be switched to the biosimilar.
		The new procurement cycle awarded adalimumab biosimilar Hulio^®^, listed at €237.8 per unit.
Netherlands	October 2018: €495.26 per unit (€990.51 per package of 2). The average reimbursement price to hospitals was approximately 80% of the listed price. Hospitals were expected to negotiate the difference in confidential discounts.	The list price is unchanged and is the same for biosimilars, but discounts of 80–90% have been reported for originator and biosimilar adalimumab (reducing prices to approximately €50–€100 per unit). Depending on the hospital. procurement group, either the originator or biosimilar is the least expensive product.
Norway	Since May 2015 to end of September 2018: €539.93 per unit (€1,079.86 per package of 2).	Now priced at €531.26 per unit (€1,062.51 per package of 2).
	Further discounts and/or rebates occurred, but are confidential. In general, the national procurement trust in Norway is able to reduce drug prices in the hospital sector with around 30%, all over. In the field of biologicals these rebates have been even larger, but the exact rebate for Humira^®^ is not public (as for other drugs).	There are additional discounts and/or rebates, but these are confidential. In press reports, discounts of 80% are reported. According to the tender recommendations for 2019, the originator is the least expensive product.
Poland	August 2018 to February 2019: €511.81 per unit (€1,023.62 per package of 2)	March 2019 to July 2019: €302.36 per unit (€604.71 per package of 2).
	Actual price: Approximately €389 per unit (August to October 2018).	Actual prices: €389.93 per unit (Nov/18), €387.42 per unit (Dec/18), €261.02 per unit (Jan/19), €233.57 per unit (Feb/19), €122.41 per unit (March/19).
		Biosimilars are listed at: €246.62 per unit (Imraldi^®^), €246.09 per unit (Amgevita^®^), €239.41 per unit (Hyrimoz^®^).
		The actual price of Imraldi^®^ was €112.60 per unit in Feb-March 2019. For this period very limited sales were observed (14 packages with 2 pre-filled syringes), however, actual prices for Imraldi^®^ were only available from February 2019.
Romania	Retail price with 9% VAT: €408.19 per unit (€816.37 per package of 2); wholesale price without VAT: €370.82 per unit (€741.63 per package of 2).	Retail price with 9% VAT: €412.38 per unit (€824.75 per package of 2); wholesale price without VAT: €374.66 per unit (€749.31 per package of 2). Prices increased a little, due to the annual update with the new exchange rate.
	No publicly-known further discounts and/or rebates existed.	The marketing authorization holder has the possibility to cover patient co-payment (which is around 26% of the retail price with VAT) through a patient access mechanism, based on an agreement implemented by an independent third party.
		The biosimilar Hulio^®^ is listed at €255.04 per unit.
Russia	2015–2018: The price for Humira^®^ according to the essential drug list (EDL) was €479 per unit (€958 for a package of 2).	2019: €414 per unit (€828 for a package of 2).
	February 2018: €411.5 per unit (€823 for a package of 2). The decrease in price can be linked to the submission of the marketing authorization dossier of the biosimilar to the ministry of health.	Discounts and rebates are confidential. The final price at the retail pharmacy level ranges from €788–€1,104 for the same package; a 5% decrease or 32% increase comparing with EDL price before any mark-ups.
	In addition to the EDL price, there is local (regional) taxation and wholesale mark-up. Those taxation and mark-up provide up to 15–25% increase of final price at the pharmacy depending on the region, creating regional price differences.	The biosimilar (Dalibra^®^) is listed at a lower price, €329.25 per unit (€658.5 for a package of 2).
	Discounts and rebates occur at the wholesale level, but are confidential. The final price at retail pharmacy has the range €788–€1,104 for the same package. Thus a 5% decrease or 32% increase compared with the EDL price before any markps.	
Scotland	€413.32 per unit (€826.64 per package of 2).	There has been no change in list price since loss of exclusivity. The actual Humira^®^ price has significantly reduced, however, there are biosimilar products that are priced lower and therefore Health Boards are in the process of switching from Humira^®^ to biosimilars. The detailed discounts are confidential.
	There was a small discount available (confidential).	
Serbia	In 2015 and 2016: €426.94 per unit. In 2017 and 2018: €391.05 per unit.	In 2019: €366.71 per unit.
	Information on discounts and/or rebates was not available.	While Humira^®^ injections are fully reimbursed, the Humira^®^ pen is currently not reimbursed in the Republic of Serbia. The Humira^®^ pen was granted marketing authorization in Serbia in February 2018 and is listed at €336.18 per unit (but not reimbursed).
		There are additional discounts in terms of providing free-of-charge medicine for 3 months’ therapy; only for new patients eligible for therapy in the first year of therapy. This information is not publicly available.
Slovenia	In 2016: €465.55 per unit (€931.09 per package of 2).	The list price has been unchanged.
	In 2017: €349.36 per unit (€698.71 per package of 2).	Further confidential discounts exist.
	Up to July 14, 2019: €335.5 per unit (€671 per package of 2).	
	There were additional discounts.	Biosimilars are listed at a lower price.
Spain	2012 – September 2019: €563.79 per unit.	The list price has been unchanged.
	There were confidential discounts.	October 2019: €326 per unit (to match the reference price established in September 2019). The originator and biosimilar have the same list price as of October 1, 2019.
		Further confidential discounts exist.
Srpska	Actual prices under contract (Health insurance fund procurement for all hospitals):.	The current actual price is still €399.29 per unit. There are no additional discounts/rebates.
	In 2014 and 2015: €561.46 per unit.	A decision on the choice of the best bidder is announced on the HIF website after completion of the tender procedure.
	In 2016 and 2017: €505.31 per unit.	Biosimilars are not yet available and reimbursed.
	In 2018: €479.08 per unit.	
	In 2019: €399.29 per unit.	
Sweden	September 2018: €462.66 per unit (€925.32 per package of 2).	May 2019: €290.32 per unit.
	A national managed entry agreement with confidential rebates exists for subcutaneous TNF-α inhibitors.	The national managed entry agreement is still in place. Different regions in Sweden currently recommend either biosimilar adalimumab or originator Humira^®^ depending on the level of discounts and other factors. For instance, AbbVie is currently offering a mix of retroactive rebates and some adjustments of its list price to help retain market share where possible with the total discount level for Humira^®^ remaining confidential. As a result, the Stockholm region has chosen, for the time being, to recommend continued use of Humira^®^ as this is currently economically the most beneficial strategy for the region.

In most countries, additional (confidential) discounts and/or rebates existed before loss of exclusivity. Exceptions included Albania, Austria, Finland, Greece, Iceland and Romania.

#### Situation After Loss of Exclusivity

After loss of exclusivity, a larger variation in list prices can be observed for originator adalimumab in the countries surveyed than before loss of exclusivity (Table 4). List prices ranged from €233.65 per unit in Austria to €955.47 in Germany (factor 4.1 difference).

**FIGURE 1:**
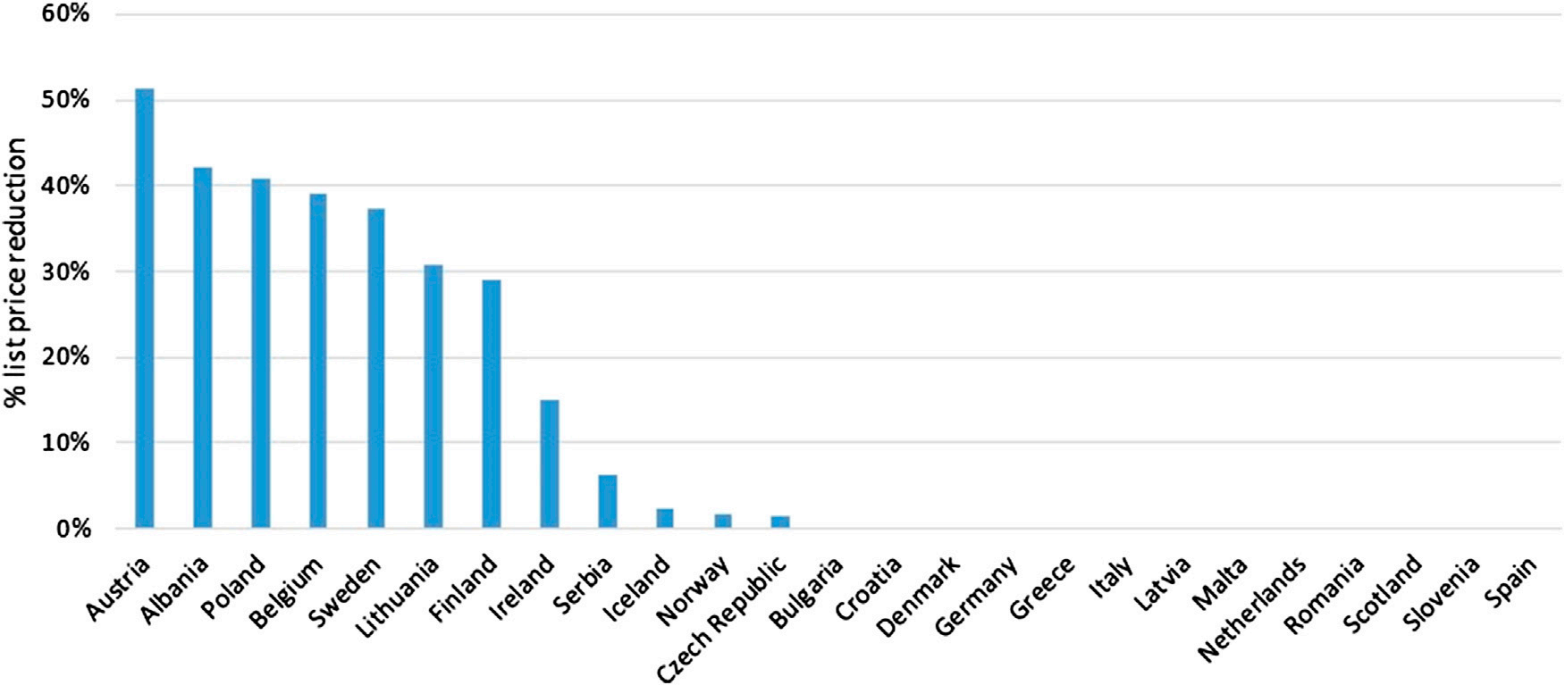
List price reductions of originator adalimumab on May 10 2019 versus list prices before loss of exclusivity (October 2018 or earlier) for all countries surveyed where list prices before and after loss of exclusivity of originator adalimumab were reported. Countries are sorted from high to low relative list price reduction (51% in Austria to 0% in several countries).

Overall, list prices of originator adalimumab (40 mg syringe or pen) decreased after loss of exclusivity rights in October 2018, and the entry of biosimilars to the market, from an average of €516 to €456 per unit (median of €430.11 per unit). For some countries, it was reported that list prices have remained unchanged up to May 2019, however, (confidential) discounts and/or rebates can exist (Bulgaria, Croatia, Denmark, Germany, Greece, Italy, Latvia, Malta, Netherlands, Romania, Scotland, Slovenia and Spain). List price decreases ranged from 0% in the previously mentioned countries to 51% in Austria, with an average list price decrease of 12% and a median decrease of 0% (Figure 1). These list price decreases may also be the result of price regulations rather than real competition.

**FIGURE 2:**
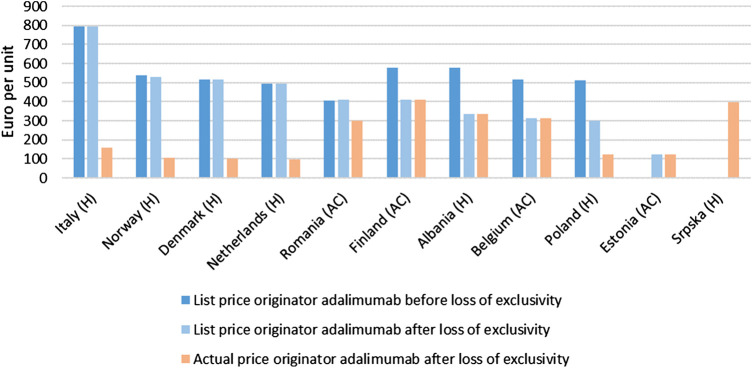
Originator adalimumab list prices (in euro per unit) before loss of exclusivity (October 2018 or earlier) and originator adalimumab list prices and actual prices after loss of exclusivity (May 10, 2019) in the countries surveyed where actual prices were reported. Countries are grouped based on whether there was a change in list price. For Estonia no list price before loss of exclusivity was reported. For the Republic of Srpska no list prices were reported. H: hospital setting; AC: ambulatory care (retail).

Furthermore, in most countries additional (confidential) discounts and/or rebates also existed after loss of exclusivity. Exceptions included Albania, Belgium, Estonia, Finland and Greece. However, Albania, Belgium and Finland had substantial decreases in list price (Figure 1). In Estonia, adalimumab had changed treatment setting (hospital to retail).

An appreciation of actual prices or indicative discounts/rebates after loss of exclusivity was obtained for Albania, Belgium, Denmark, Estonia, Finland, Italy, Netherlands, Norway, Poland, Romania and the Republic of Srpska (11 out of the 30 countries surveyed). Actual prices in these countries differed by a factor of 4.2. Figure 1 visualizes these actual prices and shows that countries that still have relatively high *list* prices after loss of exclusivity often have relatively low *actual* prices (e.g., Italy, Norway, Denmark, Netherlands). Discounts/rebates ranged from 0% (actual price same as list price) to approximately 26% (Romania), 60% (Poland), 80% (Denmark, Veneto region Italy, Norway), and 80%–90% (hospitals in the Netherlands) of the list price. In the latter countries, actual prices for one unit of originator adalimumab were approximately €301, €122.41, €104, €160, €106, and €50 – €99, respectively.

Often biosimilars are listed at the same or a lower price than the originator product and may be the least expensive product to use according to actual prices. The reverse is the exception, and specific examples, based on actual prices, where originator adalimumab was the least expensive product to use, are Norway, the Stockholm region (Sweden), and some hospitals in the Netherlands.

### Policy Measures

In addition to specific supply-side policy measures related to pricing and reimbursement that were discussed earlier, demand-side policy measures and practices might also influence originator/biosimilar market dynamics. Large variations can be seen in the efforts of countries to leverage competition from biosimilars and implement policy measures related to the use of (biosimilar) adalimumab, see Table 5.

**TABLE 5:** Policy measures related to the use of (biosimilar) adalimumab.

Country	Strategies		Aids to implementation	Outcomes
	Specific measures	Demand-side policies		
Austria	—	Recommendations (insurance funds)	• Targeted information provided to prescribers	Competitive pricing keeps the market share of originator adalimumab high
			• Recommendation to replace originator adalimumab with the more cost-effective biosimilars	
			• Information on subsequent price reductions disseminated (including price comparisons and market dynamics)	
Belgium	• Annual targets (non-binding) • Financial incentive	—	Depending on the annual target reached (5%, 10% or 20% of prescribed adalimumab and etanercept biosimilars), an amount of €750, €1,000 or €1,500 is paid	Minimal success (adalimumab biosimilars = 7.1% market share in June 2019)
Bulgaria	—	Internal referencing pricing	• Patient co-payments may influence physician’s decision to switch	May encourage more physicians to switch
Denmark	• Multi-winner tender	- Guidelines	• Therapeutic guidelines introduced (2–3 years before implementation in Nov 2018)	>90% of patients switched to biosimilar 3 weeks after implementation
			• Statement to doctors to prescribe originator for no longer than 3 months (Aug 2018)	
	• Alignment of stakeholders		Letter to patients to inform about switching (Sep 2018)	
			• Nurses present during implementation	
Estonia	—	Prescribing by INN (since July 2019)	Pharmacies dispense the least expensive product	—
Finland	—	Prescribing of least expensive product	• Physicians obliged to prescribe least expensive product	Minimal success
			• Have to justify selection of more expensive alternative in medical records	
Germany	—	• Target agreements (government)	• Target agreements per region (government)	—
		• Guidelines (government)	• Economic prescribing (government)	
		• Rebate controls (insurance companies)	• Rebate contracts with manufacturers (insurance companies)	
Greece	—	Guidelines	Economic prescribing	Greece, however, did not have any adalimumab biosimilars on the market at the time of the study
Iceland	—	Guidelines	Use of adalimumab managed by one specific hospital (Landspitali University Hospital) following clinical guidelines for each indication	—
Ireland	—	Best value biological policy	Recommendation to use the best value biologicals	—
Italy	—	Guidelines/recommendations	Communication with physicians on purchasing and formulary decisions	—
Latvia	—	• Internal referencing pricing	• Incentive of patient co-payments almost disappeared due to discount on originator adalimumab that reduced co-payments	Co-payments on originator minimal – no economic incentive to switch
			• Mandatory INN prescribing for new patients	
		• INN prescribing	• INN prescribing also allowed for patients already being treated	
Lithuania	—	Treatment guidelines	New patients prescribed the least expensive TNF-α inhibitor	—
Malta	—	• Tendering	Only one adalimumab product is procured by the national health services for the whole country (for the most recent cycle this was the biosimilar)	Takes advantage of competition
		• Negotiation		
Norway	—	Tendering	Recommend the use of originator adalimumab after Humira^®^ won the national tender	—
Romania	—	Biosimilar fully reimbursed	Patient co-payment for originator but not biosimilar. However, patient co-payment waiver possible for originator but have to re-apply every 3 months	—
Scotland	• Multi-stakeholder approach	—	• Etanercept case study made available to increase awareness	Very few patients have requested to stay on the originator
	• Switching plans		• Additional staffing	
			• Patient training on new device	
			• Sharing of statistics on biosimilar uptake	
			• Treatment cost comparator shared with Health Boards	
Spain	—	• Target agreements for biosimilars	•Target agreements for all hospitals in Catalonia that are financed by the public health system	In December 2019, only 26% of patients on adalimumab are treated with a biosimilar
		• Tendering	•Computer systems have been implemented by some regional health services in order to allow monitoring of the use of biosimilars in the hospitals	
		• Pharmacotherapeutic recommendations		
Sweden	—	Guidelines/recommendations	Communication with physicians on purchasing and formulary decisions	—
Netherlands	• Grants	—	Measures provided by insurance companies	Prevent originator monopoly and encourage competition
	• Higher reimbursement			
England	Multi-winner tender	Commissioning framework for biological medicines (including biosimilar medicines)	Contracts with different size of each lot are awarded by NHS England	Competition stimulated

Sometimes a strategy was implemented even before loss of exclusivity, for example in Denmark and Scotland. In Denmark, preparations included statements to physicians on switching, letters to patients and ensuring the presence of additional nurses to support switching. A multi-winner tender for adalimumab was chosen to ensure sustainability of the market (sales are generated for companies and shortages are prevented). The implementation of the tender relies on a well-established system of guidelines for specific therapeutic areas. Competition is enhanced because companies know that physicians will prescribe the winning product. When a company/product does not win the tender then this product can still be prescribed, but is assigned lower in the recommended ranking. Guidelines make it easy to communicate on products of first choice. During the first 3 weeks after implementation, more than 90% of patients were using biosimilar adalimumab (Amgros, 2019). In Scotland, the 14 regional National Health Service (NHS) Health Boards were encouraged to put in place plans to switch patients to biosimilar adalimumab, even before prices were confirmed. A case study on the earlier switch to biosimilar etanercept (also a subcutaneous TNF-α inhibitor) was made available on the website of Healthcare Improvement Scotland, part of the Scottish NHS, to build on the awareness, experience and clinician confidence obtained from the etanercept switch (NHS, 2016). A multi-stakeholder approach in establishing switching plans was encouraged, with patient training on the new device and additional staffing to support switching (‘invest to save principle’). Since expiry of adalimumab market exclusivity, biosimilar uptake statistics per Health Board have been shared monthly with all Boards to allow them to benchmark their biosimilar uptake against other Boards. A biological medicines treatment cost comparator for TNF-α inhibitors and competing biologicals was also developed nationally and shared with Health Boards, showing average treatment costs of different agents over a three-year time horizon. Health Boards were encouraged to review the place of individual medicines in formularies/guidelines, based on for example Multiple Technology Appraisals by the National Institute for Health and Care Excellence (NICE). For one Health Board (Greater Glasgow and Clyde), groups within gastroenterology, rheumatology and dermatology, dedicated to the efficient use of high-cost medicines, were tasked to come up with a strategy for biosimilar use. It was noticed that the regions of North Glasgow and Clyde, which already had additional staffing for rheumatology for the etanercept switch, were able to quickly switch to the selected adalimumab biosimilar with high switching rates (near 100%). In South Glasgow, where staffing had not been approved, or for specialties where etanercept is not used (e.g., gastroenterology), much lower rates of uptake were observed. Progress is monitored to switch patients to the biosimilar at their next prescription with the patient’s agreement, and has been similar or better than previous switches, confirming the validity of the process. Very few patients have requested to stay on originator adalimumab after a discussion with Health Board staff. In Italy and Sweden, regional guidelines/recommendations allow communication with physicians on purchasing and formulary decisions. In Ireland, the implementation of a best value biological policy, resulted in a strong recommendation to use the best value biologicals Imraldi (biosimilar adalimumab) or Benepali^®^ (biosimilar etanercept) when initiating treatment and issuing a repeat prescription. Interestingly, NHS England has awarded contracts to a number of manufacturers to further stimulate competition in the biosimilar adalimumab market (Davio, 2018).

In Belgium, a pilot project (started in 2019) is testing a financial incentive that encourages physicians to prescribe a percentage of biosimilar adalimumab or etanercept in ambulatory care. The objective is to compensate for the time/effort of individual physicians to switch treatment, however, so far, the success has been minimal, with various stakeholders complaining that, on the one hand, they do not agree with financial incentives to support specific products and, on the other hand, that the amount paid is too small (personal communication). Other countries also use target agreements to stimulate physicians, e.g., in Germany (per region) and in Spain (for example for all hospitals in Catalonia that are financed by the public health system). In Lithuania, physicians are obliged by treatment guidelines, which are approved by order of the Ministry of Health, to prescribe new patients the least expensive TNF-α inhibitor. For some indications, this is biosimilar adalimumab.

Insurance companies can also take measures related to the use of adalimumab. In the Netherlands, some insurance companies support hospitals which have a less favourable biosimilar contract by giving a grant for the implementation of a biosimilar or providing higher reimbursement to the hospital, which would otherwise make a loss on dispensing biosimilars. In Germany and Austria, health insurance funds use rebate contracts and non-binding recommendations. In Austria, information on subsequent price reductions of originator adalimumab and entry of new adalimumab biosimilars (with successive price reductions) was also disseminated regularly, including price comparisons and market dynamics in the wider TNF-α inhibitor class. Prescribing of the most cost-effective TNF-α inhibitor, adalimumab, was requested. However, competitive pricing keeps the market shares of originator adalimumab high (personal communication).

Physicians might be conscious of increases in patient co-payment and change treatment to reduce the financial burden on their patients. In Bulgaria, the internal reference pricing system might influence a physician’s decision and convince him/her to switch from originator to biosimilar adalimumab to avoid patient co-payment. Switching is a practice that Bulgarian physicians tended to avoid. In Latvia, this economic incentive almost disappeared due to a confidential discount for originator adalimumab after which co-payment was lowered and is only minimal. In Romania, co-payment for originator adalimumab is covered via a Patient Access Scheme, but in order to receive a co-payment waiver, patients need to go through an administrative process every 3 months. The biosimilar on the other hand is fully reimbursed without additional administrative hurdles.

Other countries did not report specific measures targeting adalimumab, but more general measures regarding biosimilars might also affect the use of adalimumab biosimilars. Often, the decision to prescribe a biosimilar is left to the physician. There might be therapeutic protocols for a disease area or national/regional guidelines. Some examples of countries with guidelines on economic prescribing include Greece, Belgium, and Germany. In Estonia and Latvia, INN prescribing ensures that pharmacies dispense the least expensive product. In Finland, physicians are obliged to prescribe the least expensive product when biosimilars are available, or justify the selection of a more expensive alternative in the medical records. In Iceland, the use of adalimumab is managed centrally, as is the case for other expensive hospital medicines. Malta reported that via tendering and negotiation it is possible to take advantage of competition.

## Discussion

This study started with the aim of gaining more insight into the competition strategies that AbbVie has pursued to protect the market share of Humira^®^, especially related to new price dynamics since the entry of adalimumab biosimilars. Although this information is not easily elucidated, we at least partly succeeded.

To the best of the authors’ knowledge, this is the first article that presents a comprehensive overview of originator adalimumab prices before and after loss of exclusivity with actual prices following loss of exclusivity obtained for 11 of the 30 countries surveyed. Countries with available and reimbursed biosimilars on the market seem to have price regulation and/or competition leading to lowered list or actual prices. This study emphasizes, once again, the importance of working with actual prices rather than list prices when studying price dynamics in the off-patent market (Moorkens et al., 2019a). We also show that the entry of biosimilars can lead to changes in the reimbursement status of a molecule/product, although few countries were able to make these changes. Additionally, this study adds to the scarce literature on policy measures affecting biosimilar/originator market dynamics (Renwick et al., 2016; Moorkens et al., 2017; Rémuzat et al., 2017a; Rémuzat et al., 2017b; Moorkens et al., 2019a; Moorkens et al., 2019b). A minority of countries implemented specific policies and practices to leverage competition following the entry of adalimumab biosimilars. However, a more general policy framework for biosimilars might have already been implemented.

A recent analysis by Vogler et al. (Vogler et al., 2019) on price developments for several molecules, including adalimumab, has shown that the average cost per milligram of a molecule decreased after the entry of the biosimilar in 15 European countries. The analysis made use of ex-factory prices up to December 2018 and combines the price of the originator with the often lower-priced biosimilar products to make an average. Our study reports list prices per product, i.e. originator adalimumab and, when reported, also for biosimilars. Additionally, it also presents actual prices to better understand market dynamics. Moreover, adalimumab price developments are included up to May 2019 to better reflect any evolution.

Our previous paper on biosimilar policies in Europe, in collaboration with the Piperska Group, illustrates the general picture for all available biosimilars up to the end of April 2017. It concluded that supply-side policies were in place in most of the countries surveyed, but that more could be done to communicate on biosimilars and educate stakeholders, especially physicians (Moorkens et al., 2017). The current paper, with information on adalimumab products up to May 2019, found that, in addition to a focus on supply-side policies, several new initiatives affecting healthcare providers have been set up. Examples of such initiatives included a best value biological policy in Ireland, a financial incentive for physicians in Belgium, additional staffing to support the switch in Denmark and Scotland, sharing previous switch plans in Scotland, and recommendations/guidelines issued in several countries to communicate on biosimilars.

Based on actual prices available for some of the countries surveyed, tendering seems to effectively drive down prices (e.g., in the hospital setting in Denmark, Italy, the Netherlands, Norway). In addition, companies marketing biosimilars are playing the pricing game, since country representatives often reported that, even with large discounts/rebates for originator adalimumab, the biosimilar is the least expensive product to use (e.g., Denmark, Italy, Poland, Romania, and some hospitals in the Netherlands). Although the size of the discounts given in the countries surveyed is often not disclosed, we have the impression that there is fierce price competition between originator and biosimilar products. This is particularly welcomed among CEE countries to help enhance the use of biologicals from a lower base compared to Western European countries. However, whilst cost decreases are certainly welcomed by health authorities and payers, as well as patients where there are high co-payment levels, a negative spiral of price decreases might undermine the long-term viability of the off-patent biologicals’ market with players opting out. In addition to list prices and actual prices shown in Table 4, claw-backs might also play a role in further lowering prices; for example in Belgium, where sales of biosimilars and low-priced biologicals (i.e., biological products decreased to the price level of the biosimilar) were at the time of the study included when calculating claw-back taxes. This is also the case in Greece and Romania. In fact, the claw-back tax for Romania in quarter two of 2019 was 25.21% (applied to the retail price without VAT), further lowering the price of originator adalimumab to approximately 50% of the list price. Earlier papers have also suggested a focus on demand-side policies, such as specific stakeholder incentives, guidelines and education, rather than solely targeting price is important to enhance their use and attain target savings (Moorkens et al., 2017; Rémuzat et al., 2017b). Target utilization patterns and savings are unlikely without a dual approach (Kim et al., 2020). This dual approach is important to help address discounting tactics by originator companies just before or soon after patent expiry to dissuade biosimilar manufacturers from entry.

Mandatory price decreases, a policy measure often seen in the retail setting and for example occurring in Austria, Belgium and Ireland, might be effective in short-term cost containment, but are probably a factor hindering competition by limiting price differences and thus incentives to switch. Market shares for originator adalimumab are still high in these countries. Alongside this, internal reference pricing and decreasing the reference price to the level of the lower-priced biosimilar, which is used in the Czech Republic, Latvia and Romania, does not always provide an incentive to use the biosimilar, as patient co-payments for the more expensive originator product can be lowered after confidential price reductions offered by the marketing authorization holder. The originator product can also decrease in list price to match the reference price (e.g., in Spain).

The price of a product and potential discounts/rebates that a company will give are also linked to the volume that can be secured. This can be dependent on the level of tendering (national, regional, hospital) and guidelines to implement a potential switch and ensure sufficient market share. For example, via its system of guidelines, Denmark is able to quickly communicate on new tender agreements. However, a multi-stakeholder approach with adequate preparation is needed to make this work.

Although the initial driver for change might be potential cost savings, other incentives provided by companies, such as research grants, patient support programs and other types of informal support, will also influence prescribers. As these initiatives are most likely already ongoing for originator adalimumab, these provide additional hurdles to overcome when changing to the biosimilar (Moorkens et al., 2016).

This study also indicated that (originator) adalimumab is reimbursed in all of the countries surveyed (except for Kosovo where it is not marketed). However, reimbursement does not always equal actual patient access to treatment, especially in some CEE countries (Kawalec et al., 2017). Hidden access barriers, such as availability only in selected patient populations and treatment centers (possibly demanding considerable efforts and cost for traveling), molecular diagnostics that are not reimbursed, waiting lists, an administrative burden to initiate therapy, and other volume restrictions have been pointed out (Mihajlo, 2014; Inotai and Kalo, 2019).

The main limitation of this study is that primarily list prices were used in the analysis rather than actual prices, impeding a real comparison between countries. However, it might still be of value to provide an overview of available information as a starting point. Prices could also be compared at the ex-factory price level, as taxes and margins differ between countries, but it was our intention to focus on expenses for the healthcare payer (even though cost per unit is not the same as expenditure). We chose to include price changes relative to the price before loss of exclusivity of Humira^®^ in October 2018. However, this could also be conducted both before and after the date of introduction of the biosimilar, which might better reflect market dynamics. As launch dates differ between countries, choosing a fixed point in time for each country (October 2018) enhances comparability of the results. Whilst the majority of data gathered via the survey was objective information regarding, for example, the availability of products and prices available in official databases, we also questioned (policy) measures related to the use of adalimumab. It is possible that the senior-level co-authors might not always know about the various measures and initiatives, especially local practices. This is the case with any research project of this nature.

The major learning from this research is that both originator and biosimilar companies adjust their strategy and pricing to each individual market, each with its own peculiarities. The result is an inexplicable pattern of prices and policies and an absence of coherence from the demand-side of the market.

Future research could focus on achieving more insight into discounts and rebates in the off-patent market by, for example, carrying out anonymous surveys/interviews; a methodology that was employed previously in studies by Vogler and colleagues (Vogler et al., 2013). In addition, the link between the development of adalimumab biosimilar market shares in different countries and actual price differences between originator and biosimilar products or other policy measures could be further investigated to help understand originator/biosimilar market dynamics, as was previously studied for infliximab and etanercept in the Swedish context (Moorkens et al., 2019a; Moorkens et al., 2019b). Furthermore, evolving volumes, market shares and policy measures could be studied together for the whole class of TNF-α inhibitors.

## Conclusion

In this paper, we document how European countries responded differently to expiry of market exclusivity on originator adalimumab and biosimilar market entry, with implications for pricing and reimbursement. Originator adalimumab list prices differed between countries by a factor of 2.8 before and a factor of 4.1 after loss of exclusivity. In most of the countries, adalimumab biosimilars entered the market and are reimbursed. However, few countries have made changes to the reimbursement status of (originator) adalimumab or implemented specific policies and practices to leverage competition following the entry of biosimilars. It seems that countries with biosimilars on the market have price competition leading to lowered list or actual prices. Reported discounts varied widely between countries.

## Data Availability Statement

The original contributions presented in the study are included in the article/Supplementary Material, further inquiries can be directed to the corresponding author.

## Author Contributions

ArV and BG developed the idea for this study and were involved in the construction of the questionnaire and critically revised the manuscript. BG was also involved in data collection. EM was involved in construction of the questionnaire, data collection and validation, and drafted the initial version of the manuscript. IsH critically revised the manuscript. IrH and AM provided data on Albania and critically revised the manuscript. SK, SS, and SM provided data on Austria and critically revised the manuscript. MD and KT provided data on Bulgaria and critically revised the manuscript. LV and VP provided data on Croatia and critically revised the manuscript. GA provided data on Cyprus and critically revised the manuscript. JS, LP, and KK provided data on the Czech Republic and critically revised the manuscript. DB provided data on Denmark and critically revised the manuscript. OL provided data on Estonia and critically revised the manuscript. JM provided data on Finland and critically revised the manuscript. GS provided data on Germany and critically revised the manuscript. VK provided data on Greece and critically revised the manuscript. EM and RE provided data on Iceland and critically revised the manuscript. RA provided data on Ireland and critically revised the manuscript. RJ and EA provided data on Italy and critically revised the manuscript. AJ provided data on Kosovo and critically revised the manuscript. AnV provided data on Latvia and critically revised the manuscript. IG-K provided data on Lithuania and critically revised the manuscript. PB provided data on Malta and critically revised the manuscript. VS provided data on Netherlands and critically revised the manuscript. ØM provided data on Norway and critically revised the manuscript. RP provided data on Poland and critically revised the manuscript. IM provided data on Romania and critically revised the manuscript. DM provided data on Russia and critically revised the manuscript. TN provided data on Serbia and critically revised the manuscript. JF provided data on Slovenia and critically revised the manuscript. CZ provided data on Spain and critically revised the manuscript. VM-P and NG provided data on the Republic of Srpska and critically revised the manuscript. GB provided data on Sweden and critically revised the manuscript. RP provided data on Scotland and critically revised the manuscript. All authors read and approved the final manuscript.

## Funding

This study was supported by KU Leuven and by the Fund on Market Analysis of Biologics and Biosimilars following Loss of Exclusivity (MABEL).

## Conflict of Interest

AGV is involved in consulting, advisory work and speaking engagements for a number of companies, a.o. AbbVie, Accord, Amgen, Biogen, EGA, Pfizer/Hospira, Mundipharma, Roche, Sandoz. RP runs a consulting company and previously worked with, a.o., Abbvie, Biogen, Sandoz, Pfizer, MSD, Roche, GSK, Sanofi. IM is involved in consulting and advisory work and recently worked with I&S Hungary, Ewopharma, Genesis Pharma, GSK. RP was employed by HTA Consulting at the time of the study. TN was employed by ZEM Solutions at the time of the study.

The remaining authors declare that the research was conducted in the absence of any commercial or financial relationships that could be construed as a potential conflict of interest.
