# Supplementary material for: The Expiry of Humira® Market Exclusivity and the Entry of Adalimumab Biosimilars in Europe: An Overview of Pricing and National Policy Measures
Source: Front Pharmacol. 2021 Jan 8;11:591134. doi: 10.3389/fphar.2020.591134 (PMC7839249; doi:10.3389/fphar.2020.591134)
Supplement: Supplementary file 1 [file datasheet1.pdf]

S1 Table. General information on financing of healthcare and levels of co-payment in the different European countries surveyed.

Country	Financing	Co-payment
**Albania**	The Health Insurance Fund (HIF) is responsible for financing health services and medicines. The Fund is financed via health insurance contributions and approximately 500 000 patients, from a country population of 2.8 million, benefit from the scheme. Every year HIF updates the list of reimbursed medicines. Different lists exist for medicines reimbursed in ambulatory care and in the hospital setting.	The general level of co-payment varies from 10% to 100% for the ambulatory care and hospital lists. Humira® is present in the hospital list and is reimbursed for patients up to 68% (approximately €108 patient co-payment).
**Austria**	Healthcare in Austria is based on a social insurance model. The Austrian Social Insurance (*Dachverband der österreichischen Sozialversicherung*, DVSV) is the umbrella organization of the three main sickness funds and two further social insurance institutions. About 99.9% of Austria's population are covered by statutory social health insurance, mainly organized according to regional employment affiliation and vocational groups; there is no free choice of the sickness fund. The system is characterized by income-related health insurance contributions, benefits in kind, direct access to primary, secondary and tertiary care, with co-payments at all levels of care.<br><br>The purchasing of medicines in the hospital setting is organized in a decentralized way, with decisions taken by the individual hospital owner organizations.	In ambulatory care, medicines are granted in kind to the insured (e.g., for Humira®). There is only a single fixed prescription fee per item. This is currently €6.10 (except when the pharmacy price is lower), and is adjusted yearly. The co-payment is waived in certain cases: 1) If the medicine is for an officially recognized communicable disease, 2) If the patient is exempt, due to low income, or people with low income who have extraordinarily high expenses due to sickness or disability, 3) Persons who have already paid more than 2% of their yearly net income (at least 37 fees) are automatically exempt from the co-payment prescription fee.<br><br>In the hospital setting medicines are distributed there without further co-payment.

Country	Financing	Co-payment
**Belgium**	Medicines in Belgium are financed via taxes, compulsory health insurance (via a sickness fund of choice), and patient co-payments.	In ambulatory care, including ambulant patients in the hospital, co-payment varies from 0% to 100% based on different reimbursement categories. Co-payment for Humira® and its biosimilars is limited to €12.10 per package.<br><br>In the hospital setting, the use of medicines is included in a lump sum payment.
**Bulgaria**	In Bulgaria medicines are financed via health insurance. They are reimbursed with public funds if they are included in the Positive Drug List (decision maker – National Council on prices and reimbursement of medicines) after detailed information for safety and efficacy and HTA (for new INNs) or pharmacoeconomic analysis (for generic medicines, new dosage form, route of administration or therapeutic indication and maintenance of reimbursement status).	In ambulatory care (Annex I of the Positive Drug List), the percentage of reimbursement varies from 25% to 100%, depending on the safety, efficacy and pharmacoeconomic evaluation and the type of treatment, the social impact of the disease, the duration of treatment and the pharmaco-therapeutic guideline (e.g., Humira® 75% reimbursement). Note: Internal reference pricing is applied as a cost-containment measure, so that actually the National Health Insurance Fund (NHIF) pays a certain percentage of reimbursement from the reference price.<br>In the hospital setting, (Annex II of the Positive Drug List), the percentage of reimbursement is 100%.<br>Biological medicines are included in Annex I, but based on the preferences of the company, they could also apply for Annex II.
**Croatia**	Medicines are financed via health insurance contributions (major part) and general taxes.	In ambulatory care, co-payment of prescription medicines is determined through therapeutic reference pricing (around 60 clusters, most at ATC 4 and 5 levels) and a fee per prescription of circa €1.5.<br>No co-payments exist for hospital care medicines (including adalimumab and other biological medicines).
**Cyprus**	This information was not reported.	This information was not reported.

Country	Financing	Co-payment
**Czech Rep.**	Medicines are financed via health insurance contributions.	In ambulatory care, co-payment is given by the difference between the pharmacy retail price and the maximum reimbursement price (for all drugs, including biologicals such as adalimumab). The State Institute for Drug Control (SUKL) decides on both maximum ex-factory price and reimbursement price. However, the medicine can be marketed with a lower ex-factory price - it depends on the decision of the producer, or on special contracts between producer and hospital or health insurance companies. Then, the co-payment can be lower. Note: in hospitals, both in-patient and out-patient care can be provided. Medicines that are used in hospital care (in-patient) are fully reimbursed (at the level of the least costly variant, depending on the degree and severity of the disease - without any co-payment for patients).
**Denmark**	Medicines are financed via taxes. The government pays for medicines in the ambulatory care setting and the regions pay for medicines in the hospital setting.	In ambulatory care, co-payment exists. In the hospital setting (e.g., adalimumab and other expensive medicines), medicines are free for the patient.
**Estonia**	Medicines are financed from the social security tax based on solidarity. There is one payer in Estonia – the Estonian Health Insurance Fund. Adalimumab is financed by the Estonian Health Insurance and is delivered via hospital pharmacies as a part of healthcare service. From July 2019 it is also dispensed in general pharmacies as a regular reimbursed medicine.	In ambulatory care, there are three different levels of reimbursement – 50%, 75% (90% for the elderly) and 100%. The diseases are classified in different reimbursement categories based on severity. Patients have to pay a €2.50 prescription fee and the remainder of the price of the medicine according to the reimbursement level (0% co-payment for adalimumab). If the patient prefers a product that is more expensive than the current cheapest one, the patient has to pay the extra cost out of pocket.<br>In hospital care there is no co-payment.

Country	Financing	Co-payment
**Finland**	Finland has two sources of public financing for health services: municipal financing based on taxes and National Health Insurance (NHI) based on compulsory insurance fees. Municipalities fund medicines used in hospitals and NHI funds outpatient medicines.	In ambulatory care (e.g., adalimumab), reimbursements for prescription medicines are available only after meeting an initial deductible of €50 per calendar year. Children and adolescents (aged under 18 years) are exempt from the initial deductible. Three reimbursement categories exist: the Basic Refund Category (reimbursement 40%; copayment 60%), and the Lower (reimbursement 65%; copayment 35%, for example for adalimumab) and Higher (copayment of €4.50 per medicine and per purchase; rest is reimbursed by 100%) Special Refund Categories. The categories are graded according to medical criteria based on the severity of the disease and the necessity of the drug treatment. An annual threshold of €572 exists. When it is exceeded, the patient receives additional refund (only €2.5 co-payment per medicine for the rest of the year).<br>In the hospital setting, no separate fee is charged on medicines. A patient only pays a client fee for the interventions during the hospital stay. Medicines administered during the visit are included in this fee.
**Germany**	Medicines are financed via statutory health insurance for circa 90% of the population; others are privately insured and/or have state benefits (e.g., soldiers, police, and jail inmates).	In ambulatory care, a co-payment exists of 10% of the retail price (minimum €5 to maximum €10). Exemptions apply for children below 12 (longer under certain conditions) and for low-income groups. This pertains to all reimbursable medicines. Co-payment for originator and biosimilar adalimumab is €10.<br>In the hospital setting, co-payment is based on the length of stay, but not on the medicine use.
**Greece**	Medicines are financed via health insurance contributions.	For special categories of diseases and medicines, including high-cost medicines and biological medicines such as adalimumab, there is no co-payment when dispensed via public pharmacies (hospital pharmacies,

Country	Financing	Co-payment
		EOPYY-pharmacies). The average co-payment for medicines delivered from private pharmacies is 24%, (14.3% standard co-payment and 9.6% additional charges).
**Iceland**	Medicines are financed via compulsory taxation.	In Iceland, medicines are classified in two groups: general medicines and hospital medicines. Adalimumab is classified as a hospital medicine and furthermore, a physician has to apply for its use via the Drug and Therapeutics Committee of the hospital. All medicines that are classified as hospital medicines in Iceland are free of charge for the patient. If there are biosimilars available, the least expensive treatment option is the first choice.
**Ireland**	Ireland's healthcare system is primarily funded by compulsory taxation. A proportion of the population does also have private healthcare insurance (40-50%) - this increased considerably following the proposal to implement universal healthcare insurance.	Ireland has a number of reimbursement schemes for medicines dispensed in the community setting: the General Medical Scheme (GMS), Long Term Illness Scheme and the Drugs Payment Scheme (DPS). Co-payment occurs on only the GMS and the DPS schemes. For the GMS (with a tested medical card) the co-payment is €2 per prescription item up to €20 per family per month. If the person is over 70 years the co-payment is €1.50 up to a family monthly value of €15. The DPS is available to all persons who are not eligible for a full medical card and the threshold above which a family does not have to pay is €124 per calendar month. There is an 'arrangement' for expensive medicines (under which many of the biosimilars fall) whereby if a patient has a medical card they do not have to pay an additional charge and if they have a DPS card they pay up to €124.
**Italy**	Medicines are financed via compulsory taxation.	No co-payments exist for medicines in ambulatory care and hospital setting (e.g., adalimumab).

Country	Financing	Co-payment
**Kosovo**	Compulsory taxation is used for medicines that are listed in the National Essential Medicines List (NEML). From the central budged of the country, the Ministry of Finance allocates a certain budget for the Ministry of Health to cover the cost of the medicines listed in the NEML.<br>Humira is still not marketed in Kosovo, and is also not in the NEML. From the group of TNF-α inhibitors only etanercept and infliximab are listed in the NEML.	No co-payments exist (if the medicine is in the list and is available, it is free to the patients).
**Latvia**	Medicines are financed via general taxation in Latvia.	In Latvia the reimbursement is diagnosis based. Based on the character and severity of the disease, medicines are reimbursed at 100% (e.g., adalimumab), 75% or 50% rate of the (reference) price.<br>The patient has to co-pay when the medicine is reimbursed at 75% or 50% rate, and also when not the reference (cheapest) product is prescribed, the patient pays the difference between the reimbursement sum and the price of the product, for example in the case of Humira® (but co-payment is insubstantial after an additional discount offered by AbbVie).
**Lithuania**	Medicines are financed via health insurance contributions.	In ambulatory care, all medicines are 100% reimbursed, but all medicines have a minimum co-payment (not higher than 20% of the reimbursement price for medicines with a retail price less than €20 and for more expensive medicines patient co-payment is not higher than €4.71, e.g., for originator and biosimilar adalimumab). Co-payment can be covered by the marketing authorization holder via confidential discounts.<br>Medicines in hospital care are without co-payment.

Country	Financing	Co-payment
**Malta**	The system for public healthcare is financed through taxes and through payment of national insurance. Payment of national insurance is mandatory for all workers. Relatively few people have an additional private health insurance, and the people who have private health insurance will have to pay for the private insurance additionally (over and above) the mandatory national insurance.<br><br>The medicines supplied through the national health services are all procured centrally and this stock is distributed to public hospitals and to community pharmacies (for primary care). During central procurement the cheapest available alternative is procured.	Medicines provided through the public healthcare system, such as adalimumab, are accessible totally free of charge for patients who are entitled to free medicines through the public healthcare system. Entitlement is according to a list of disease conditions as set by law.<br><br>A patient is either entitled to free treatment and gets the medicine totally free of charge (provided the medicine is supplied through the national health services and the patient is entitled to the medicine for free) or alternatively the patient has to buy the medicine privately and pay the full price for it.
**Netherlands**	Medicines are financed via health insurance contributions, except for expensive orphan drugs which are funded via a lump sum of the Ministry of Health to the healthcare insurers. Adalimumab is funded through a so called “add-on”, a financial supplement for an expensive drug. The hospital declares the add-on directly to the health insurer and it is determined on the basis of the negotiating position of the hospital and the health insurer in question. The healthcare insurer receives its funds by collecting premiums from the insured parties.	In the Netherlands there is only co-payment for a small proportion of non-essential "luxury" medicines (not good enough for general reimbursement, poor cost-benefit ratio etc.).

Country	Financing	Co-payment
**Norway**	In Norway medicines are financed from the state based on state income including taxation. The use of medicines by patients receiving treatment in the hospital is covered by the state through the specialist healthcare, which is owned by 4 regional health authorities (reporting to the Ministry of Health). In ambulatory care, for treatment of chronic conditions, the state (insurance system) will cover the major part of the cost. Private health insurance has a minor role in the Norwegian health care system, which is based on a public model.	In ambulatory care, there is 39% co-payment for medicines used for chronic treatment or long-standing conditions, with a maximum of 2369 Norwegian kroner (~235 euro) per year. In the hospital setting (e.g., adalimumab) there is no co-payment. Biological medicines are covered through a particular financing system under the specialist health care, the so-called H-prescriptions (Hospital prescriptions). All medical treatments under this system are 100% covered by the hospitals to which the patients belong, i.e. including all treatments outside the hospitals for such drugs.
**Poland**	Medicines are financed via health insurance taken from taxation.	Medicines used in the hospital and ambulatory care setting are fully reimbursed (up to the reference price). Biological medicines are used only the hospital setting, and are free of charge to patients (even when a more expensive adalimumab is used, the hospital will cover the cost above the reference price). Healthcare providers are receiving remuneration from the National Health Fund. This remuneration is limited to the cheapest one in the limit group.
**Romania**	Medicines are financed mainly via health insurance contributions, the National Health Insurance House (NHIN) dedicated budget, and partially via the Ministry of Health (only for some products included in Public Health National Programs e.g., medicines for HIV-AIDS and tuberculosis).	In ambulatory care, the reimbursement level varies between 20% and 100% of the reference price, depending on the sub-list. Originator adalimumab had first no co-payment, but this was increased to €106.3 per unit due to a change in reference price after the entry of biosimilars. However, co-payment can be partially/completely covered by the marketing authorization holder via a patient support program.<br>In the hospital setting no co-payment of medicines exists.

Country	Financing	Co-payment
**Russia**	Medicines are financed according to compulsory taxation. However, the reimbursement system is changing and moving from compulsory taxation (like it was in the Former Soviet Union) to health insurance. Insurance funds have been already established to collect money from different sources and distribute this directly to medical organizations (hospitals, etc.) or to resellers responsible for providing medicines to medical organizations. The main source of money for those insurance funds is still state budget based on compulsory taxation.	No co-payments exist (no charges for prescription medicines in any hospital or ambulatory care setting in Russia).
**Scotland**	Medicines are financed via compulsory taxation.	No co-payments exist (no charges for prescription medicines in any hospital or ambulatory care setting in Scotland).<br>There is Scottish government advice for co-payment if a medicine has not been approved by the Scottish Medicines Consortium (SMC) and a request by the Peer Approved Clinical System (PACS) process has also been rejected. If the patient still wishes to use it with the support of a private clinician then co-payment can be used. With biological medicines for autoimmune disorders this has not been necessary as no medicine has been rejected by the SMC.
**Serbia**	Healthcare (medicines as well) in Serbia are mainly financed by mandatory contributions to a social health insurance scheme. The National Health Insurance Fund (RZZO-changed to RFZO in 2011) is the major financing source of the Serbian healthcare system. RFZO is in charge of collecting contributions, pooling resources, and purchasing services from healthcare providers, of which the majority are owned by the Ministry of Health or municipal governments.<br>Mandatory health insurance premiums are levied on salaries of employees	The List of Medicines (Reimbursement List) consist of:<br>List A - medicines which are prescribed and dispensed by medical prescription (100% reimbursement, patient paying the prescription premium)<br>List A1 - medicines which have alternatives in A category (10-90% co-payment for patients)<br>List B - medicines which are for parenteral use, dispensed by healthcare institutions (medicines dispensed upon written order)

Country	Financing	Co-payment
	(employer and employee pay equal portions) as well as from farmers and the self-employed. Both employers and employees are required to contribute 5.15% of their payroll-wages to health insurance The self-employed contribute 10.3% of declared income.<br>The reimbursement list includes the medicines that are prescribed and dispensed under the mandatory health insurance scheme (at the cost of the Republic Institute of Health Insurance), as well as the conditions under which such medicines are available to insured persons.	List C - medicines with special regime of dispensing (only hospitals), for example adalimumab<br>List D - Medicines which do not have market authorization in the Republic of Serbia, and are necessary for diagnostics and therapy – non-registered medicines (in-out patient care)<br><br>Biological medicines are on List C, there is no co-payment for medicines on List C, and they are only available in the hospital.
**Slovenia**	Medicines are financed via National Health Insurance (compulsory).	With the co-insurances, which almost the whole adult population has, there is no co-payment. Co-payment is obligatory only in case of medicines that are priced higher than the reference level (reference pricing system for ATC 5&4). Adalimumab is not included in the internal reference pricing system, hence there is no co-payment.
**Spain**	Medicines are financed via compulsory taxation.	In Spain, several cases have to be differentiated:<br>a) If the patient is admitted to a public hospital, all medication received during admission will be paid by the hospital and the patient will not have to pay any co-payment. b) If the patient is not hospitalized, the doctor will write a prescription that, depending on the type of drug in question (complexity of the treatment, cost, etc.), the patient will have to pick it up either at a hospital pharmacy service (no co-payment, e.g. Humira®) or at the community pharmacy (several types of co-payment, depending on the patient's income, whether he is a pensioner of social security and whether it is a chronic treatment). Most of the biological medicines are dispensed in hospital pharmacy services and a small number of them in community pharmacies (e.g., teriparatide, insulin, denosumab etc.).

Country	Financing	Co-payment
**Srpska**	The Republic of Srpska is one of the two constitutive entities in Bosnia and Herzegovina. It has executive and legislative functional responsibilities covering healthcare policies. The authority for providing healthcare is centralized, with the Ministry of Health and Social Welfare responsible for planning, regulation and management functions.<br>Healthcare (medicines as well) is funded mainly via compulsory health insurance contributions. The Health Insurance Fund of the Republic of Srpska (HIF) provides health insurance coverage for the population, and it operates on the basis of solidarity and mutuality. HIF funds the majority of medicines in the health system via few medicines lists upon which it procures medicines (public procurement). Medicines dispensed in the pharmacy with a prescription (the List of medicines dispensed upon a prescription, so-called 'Positive List') are also funded by the HIF, but are not procured via public procurement, but are included in an internal reference pricing system.	The List of medicines dispensed upon a prescription in ambulatory care consists of a List A and a List B. List A is a basic list of medicines, and medicines are covered 100% up to the reference price level for patients exempt from the co-payment; otherwise, there is a 10% co-payment of the reimbursed reference price combined with the additional co-payment for a product more expensive than the reference price. List B is a complementary list with a mandatory 50% co-payment. Adalimumab is not on the List, i.e., cannot be dispensed in the retail pharmacy upon a prescription.<br><br>There is no co-payment for medicines used in hospitals (e.g., adalimumab), including for biological medicines.
**Sweden**	Financing of medicines in the public healthcare system occurs via taxes.	In ambulatory care (e.g., Humira®), differential co-payments exist up to 2300 Swedish kronor a year for 2019 (approximately €220), and 2350 Swedish kronor a year in 2020 (approximately €225) for all prescribed medicines in the reimbursement scheme – free after that. Children younger than 18 year do not pay any co-payment.
